# Rebuttal From Dr Kim et al

**DOI:** 10.1016/j.chpulm.2024.100069

**Published:** 2024-06-11

**Authors:** Roger Y. Kim, Catherine R. Sears, Nicholas J. Pastis

**Affiliations:** Division of Pulmonary, Allergy and Critical Care (R. Y. K.), Department of Medicine, University of Pennsylvania, Philadelphia, PA; the Division of Pulmonary, Critical Care, Sleep and Occupational Medicine (C. R. S.), Department of Medicine, Indiana University School of Medicine, Indianapolis, IN; the Division of Pulmonary Medicine (C. R. S.), Richard L. Roudebush Veterans Affairs Medical Center, Indianapolis, IN; and the Division of Pulmonary, Critical Care and Sleep Medicine (N. J. P.), Department of Internal Medicine, The Ohio State University College of Medicine, Columbus, OH.

We read with great interest the Nadig et al editorial^1^ highlighting the arguments against the clinical usage of liquid biomarkers for risk stratification of pulmonary nodules. We share their desire for large, prospective randomized controlled trial data to help guide our critical assessment of novel biomarkers and to provide the best care possible to patients with indeterminate pulmonary nodules by diagnosing lung cancer in a timely fashion while avoiding exposure to unnecessary and potentially harmful procedures. Our colleagues suggest that because definitive evidence of clinical utility is not currently available and physicians are not yet fully familiar with result interpretation, clinical implementation of commercially available lung cancer diagnostic biomarkers should not be pursued. We acknowledge that more work needs to be done to optimize implementation of novel biomarkers into routine clinical practice but disagree that no efforts should be made to harness the potential benefit of using biomarkers as complementary information to inform clinical decision-making while enhancing guideline-directed care.

The current state of pulmonary nodule risk stratification and management is inadequate, as evidenced by a lack of standardized practice among physicians and poor adherence to published guidelines.^2–5^ Moreover, patients often feel confused and anxious when cancer risk is not explicitly discussed.^6^ Therefore, it is paramount for pulmonologists to embrace well-validated tools which provide much-needed supplemental information on nodule malignancy risk to guide clinical management decisions. In the current information age landscape in which we practice, patients are often a step ahead of us regarding internet searches for the most cutting-edge technology relevant to their medical conditions and expect us to be knowledgeable in and offer them all available resources when engaging in shared decision-making.^7^ Because liquid biomarkers are already commercially available for clinical use in the United States, we as a field must learn how to best implement these tests into our clinical practice even as we continue to conduct the large-scale clinical utility trials essential to advancing our knowledge of their effectiveness.

As our colleagues have astutely pointed out, additional research, education, and training is required before physicians will broadly implement liquid biomarkers into their practice, but this does not mean that the tests themselves are not yet ready for clinical implementation. Multiple rigorous clinical validation and registry studies have demonstrated that liquid biomarkers provide a meaningful supplemental datapoint to physicians,^8,9^ and additional prospective clinical studies are already underway. It is now up to us to determine how and in what clinical situations to use this information when we discuss next steps in management with patients. If we want to improve how we manage indeterminate pulmonary nodules, perhaps rather than returning to our “regularly scheduled programming,” we should instead take inspiration from Albert Einstein, who reminds us that “insanity is doing the same thing over and over again and expecting different results.”
